# Bilateral renal lymphangioma - An incidental finding

**DOI:** 10.4103/0971-4065.65309

**Published:** 2010-04

**Authors:** S. Magu, S. Agarwal, S. K. Dalaal

**Affiliations:** Department of Radiodiagnosis and Surgery, Pt. B D Sharma, PGIMS, Rohtak, Haryana, India

Renal lymphangiectasia is a very rare benign disorder of the renal lymphatics. It is known by many different names including renal lymphangiomatosis, parapelvic lymphangiectasia, hygroma renale and polycystic disease of the renal sinus.[1] The origin is unclear and it is generally regarded as a developmental malformation of the renal lymphatic system. It may be seen at any age. Familial association has been reported in some cases.[2] It can be confused clinically, radiologically, and surgically with autosomal dominant polycystic kidney disease in adults, and autosomal recessive polycystic disease in children. However, on imaging, these cysts are large, thin walled, parapelvic and central retroperitoneal in location. Renal parenchyma is relatively normal as against the other two conditions.[2] Clinically, it is usually asymptomatic and incidentally diagnosed.[1]

The complications include hematuria, ascitis, occasional deterioration in renal function and rennin dependent hypertension.[3] The treatment of asymptomatic cases is not required. When collections are very large and causing pressure symptoms, percutaneous drainage may be carried out.[1]

**Figure 1:**
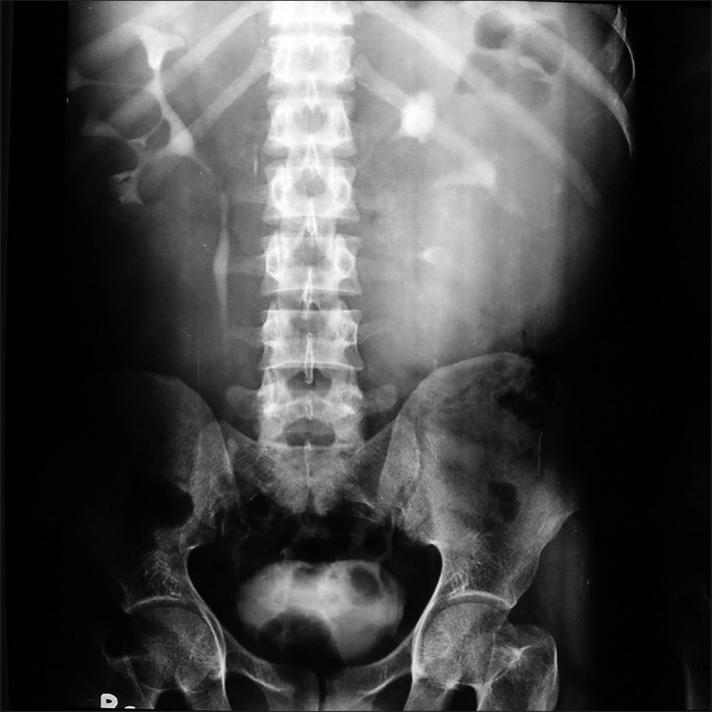
IVU image taken at 15 minutes reveals distortion of calyceal outline

We report the case of a 28-year-old male patient who presented with mild dull aching pain abdomen for the past seven years. There was no other significant history in the past. An ultrasound was performed six years back with aspiration of approximately 20 ml fluid from the lesion for diagnostic purposes and he was diagnosed with renal lymphangioma. He was put on symptomatic treatment and asked to come for regular follow-up. During the present visit his renal functions were normal. Intravenous urography (IVP) revealed normal function bilaterally. However, the calyces were distorted [Figure 1].

**Figure 2:**
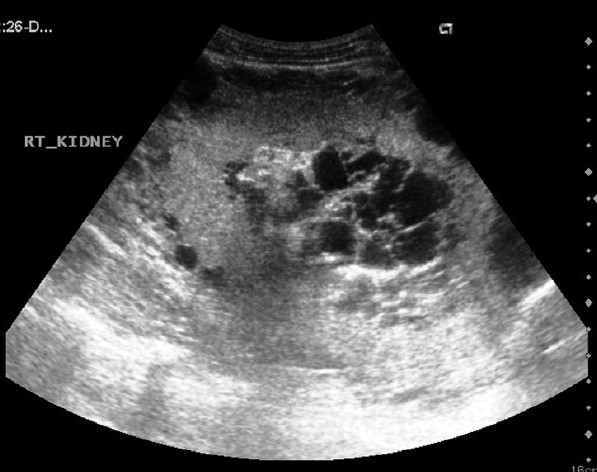
Axial USG image of the right kidney reveals multiple cysts in the perirenal region and the region of the pelvis

Ultrasonography (USG) revealed multiple cysts in the perirenal and peripelvic location, on both sides, distorting the renal outline [Figure 2].

**Figure 3:**
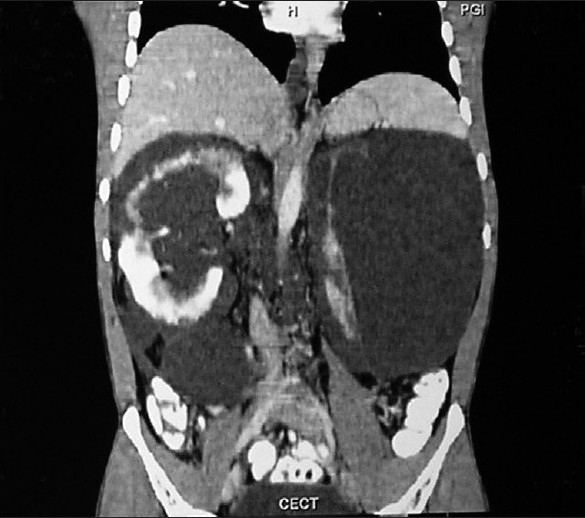
Reformatted contrast enhanced coronal CT image reveals non enhancing extra-parenchymal hypo dense cysts in the perirenal region and the region of the pelvis of both the kidneys (more on the left side) causing distortion of the underlying parenchyma without involving it

**Figure 4:**
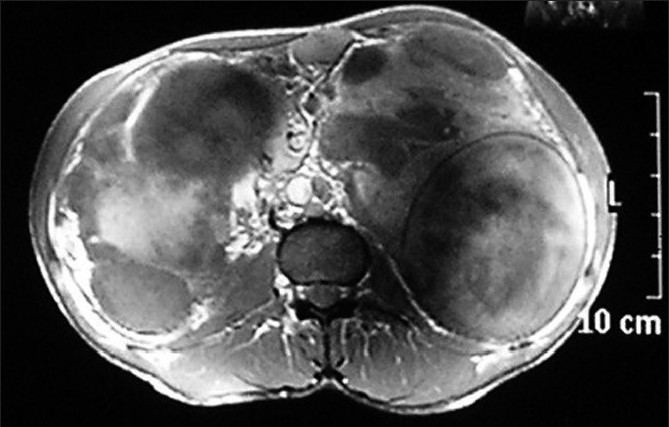
Axial non-contrast enhanced T1-W MR scan at the level of the kidneys reveals that the cysts are of hypo-intermediate signal intensity

**Figure 5:**
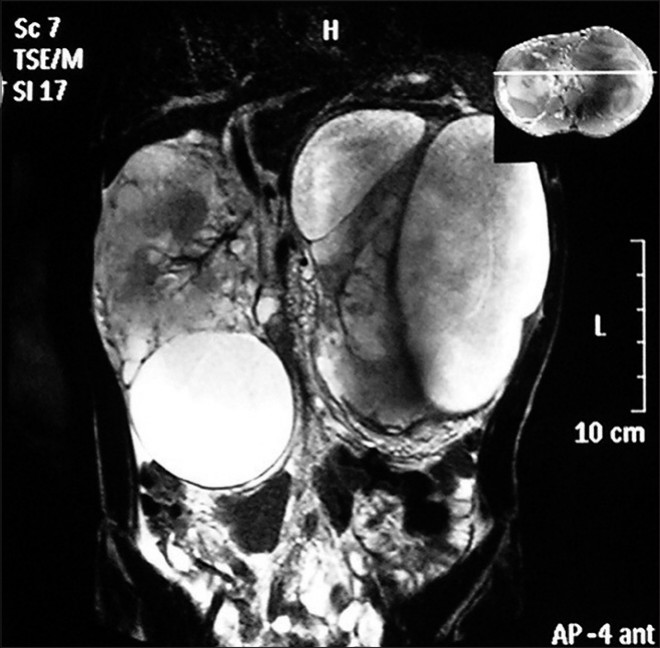
Coronal T2-W MR scan at the level of kidneys reveals that the cysts are of hyper intense signal intensity

Computed tomography (CT) and magnetic resonance imaging (MRI) revealed that the cysts were non-enhancing and were of cystic density on CT, [Figure 3] while they were of hypo-intermediate signal intensity on T1-weighted images and hyper intense on T2-weighted images [Figures 4 and 5]. On no imaging modality was the parenchyma of the kidneys involved, however, it appeared markedly reduced in volume at the present visit. In view of the apparent parenchymal compromise, approximately 600 mL of chylous fluid was drained from both sides. The aspirated fluid was rich in protein and contained rennin. On microscopic examination only a few lymphocytes were seen, while no organism was isolated on culture of the fluid.

We present this case to highlight the benign nature of this condition as against the conditions it can be confused with.
